# *Schistosoma mansoni* Infection in Ugandan Men Is Associated with Increased Abundance and Function of HIV Target Cells in Blood, but Not the Foreskin: A Cross-sectional Study

**DOI:** 10.1371/journal.pntd.0004067

**Published:** 2015-09-03

**Authors:** Jessica L. Prodger, Aloysious Ssemaganda, Ali Ssetaala, Paul K. Kitandwe, Enoch Muyanja, Juliet Mpendo, Annet Nanvubya, Mathias Wambuzi, Leslie Nielsen, Noah Kiwanuka, Rupert Kaul

**Affiliations:** 1 Departments of Medicine and Immunology, University of Toronto, Toronto, Canada; 2 Uganda Virus Research Institute-International AIDS Vaccine Initiative HIV Vaccine Program, Entebbe, Uganda; 3 International AIDS Vaccine Initiative, New York, New York, United States of America; 4 School of Public Health, College of Health Sciences, Makerere University, Kampala, Uganda; Centers for Disease Control and Prevention, UNITED STATES

## Abstract

**Background:**

*Schistosoma mansoni* infection has been associated with an increased HIV prevalence in humans and SHIV incidence in primate models. We hypothesized that immune activation from this gastrointestinal mucosa infection would increase highly HIV-susceptible CD4 T cell subsets in the blood and the foreskin through common mucosal homing.

**Methodology/Principal Findings:**

Foreskin tissue and blood were obtained from 34 HIV- and malaria-uninfected Ugandan men who volunteered for elective circumcision, 12 of whom were definitively positive for *S*. *mansoni* eggs in stool and 12 definitively negative for both *S*. *mansoni* eggs and worm antigen. Tissue and blood T cell subsets were characterized by flow cytometry and immunohistochemistry (IHC). Th17 and Th1 cells from both the blood and foreskin expressed higher levels of CCR5 and were more activated than other CD4 T cell subsets. *S*. *mansoni*-infected men had a higher frequency of systemic Th1 cells (22.9 vs. 16.5% of blood CD4 T cells, p<0.05), Th17 cells (2.3 vs. 1.5%, p<0.05), and Th22 cells (0.5 vs. 0.3%, p<0.01) than uninfected men. Additionally, Th17 cells in the blood of *S*. *mansoni*-infected men demonstrated enhanced function (28.1 vs. 16.3% producing multiple cytokines, p = 0.046). However, these immune alterations were not observed in foreskin tissue.

**Conclusions/Significance:**

*S*. *mansoni* infection was associated with an increased frequency of highly HIV-susceptible Th1, Th17 and Th22 cell subsets in the blood, but these T cell immune differences did not extend to the foreskin. *S*. *mansoni* induced changes in T cell immunology mediated through the common mucosal immune system are not likely to increase HIV susceptibility in the foreskin.

## Author Summary

Fishing communities in East Africa have a very high prevalence of HIV, and also high rates of other endemic infections such as malaria and the fluke *Schistosoma mansoni*. Genital infections are known to increase HIV susceptibility through the recruitment and activation of mucosal CD4 T cells to the site of HIV sexual exposure. These activated CD4 T cells are necessary for an effective host immune response but are also preferentially infected by HIV. We hypothesized that *S*. *mansoni* infection in the gut mucosa might increase recruitment and activation of HIV target cells at other mucosal sites, and thereby contribute to high HIV rates in fishing communities. We enrolled men from a fishing community in Uganda and examined the frequency of highly HIV-susceptible cell types in their blood and foreskin tissue (a main site of HIV acquisition in heterosexual men). We found that men with *S*. *mansoni* infection had a greater frequency of HIV target cells in their blood, but not their foreskin tissue, perhaps because foreskin cells did not express mucosal homing markers. It is possible that HIV target cells observed in the blood of *S*. *mansoni*-infected individuals may traffic to other mucosae, such as the vagina or gut, and so the possibility that *S*. *mansoni* infection increases risk at these sites should be explored.

## Introduction

HIV continues to be a public health crisis, with 2.3 million new infections and 1.6 million HIV-related deaths in 2013. Most new infections (70%) occurred in sub-Saharan Africa (SSA), where the predominant mode of transmission is heterosexual sex [1]. Despite the high number of new cases of HIV, the likelihood of transmission during a single sexual exposure is low, and is almost always established by a single virus quasispecies out of multiple distinct strains in the transmitting partner [2]. This suggests that the genital mucosa presents a significant barrier to infection. The substantial heterogeneity in susceptibility between individuals [3, 4] may reflect differences in the availability of target cells in the genital mucosa [5, 6], and increased levels of genital immune activation may account for the much higher per-contact risk of acquisition after exposure in SSA [7, 8].

CD4 T cells expressing the chemokine receptor CCR5 are the predominant targets of HIV during initial infection [2, 9, 10], and specific CD4+ T helper (Th) subsets are particularly susceptible to HIV. Activated Th cells are more susceptible to infection [11–13], as are Th17 cells (defined by the production of IL-17 [14]), Th1 cells (produce IFNγ [15]) and Th22 cells (produce IL-22 in the absence of IL-17 or IFNγ [16–18]). Not only are these subsets more susceptible to HIV infection *in vitro* [19–22], but they are also selectively depleted early in HIV infection [21, 23–25], and are less frequent in HIV-exposed seronegative (HESN) men [26]. Th17 cells have the capacity to not only produce IL-17, but also other pro-inflammatory cytokines, including IL-22 and IFNγ [27, 28]. Polyfunctional Th17 cells are more susceptible to HIV infection *in vitro* than either Th1 cells or Th17 cells that produce IL-17 alone [20–22], and are rapidly depleted in early HIV infection [28]. The mucosal availability of these highly susceptible CD4 T cells may determine whether exposure to HIV results in infection [5].

In keeping with the role of these mucosal cell subsets in HIV susceptibility, their numbers are increased in the genital mucosa by sexually-transmitted infections (STIs) that enhance HIV risk, such as Herpes simplex virus type 2 (HSV-2) [29–34], even in the absence of clinically apparent ulceration [35]. Recent studies show that non-genital infections common to SSA, such as helminthic infections, promote systemic inflammation and CCR5 expression [36–40]. Whether immune activation from infections of the gastrointestinal mucosa, such as helminthic infections, would translate into genital immune alterations is not known, but immune stimulus at one mucosal surface often leads to T cell activation and recruitment at distal mucosal sites through the expression of common mucosal homing markers [41]. This may explain why Kenyan women without STIs nonetheless have an increased frequency of activated CD4 T cells in their cervical mucosa compared to their peers from the United States [8]. While helminthic infections were not tested for in this study, their high regional prevalence suggests a potential role in the mucosal immune activation observed in these otherwise healthy women.

HIV disproportionately affects fishing communities in SSA; incidence rates on the shores of Lake Victoria in Uganda approach 5%, while the national average is under 0.75% [42–44]. Helminthic infections are very common in these fishing communities, with over 60% of men aged 20–29 years infected with *Schistosoma mansoni* [45], a parasitic worm that causes a chronic gastrointestinal (GI) infection if untreated and has been associated with increased HIV prevalence [46]. During infection, cercariae (free-swimming larval stage) found in contaminated fresh water penetrate the skin and migrate through the circulation to the portal vein where they mature into adult worms. Females release approximately 300 eggs per day, which traverse the walls of the vein and intestine to be released into the gut lumen and excreted [47]. While the worms themselves evade immune destruction [48], eggs are highly antigenic and not only require a strong CD4 T cell response for efficient release [49, 50], but also incite the formation of CD4 T cell-containing granulomas in intestinal tissue [47]. The mechanism for the potential association between *S*. *mansoni* infection and HIV susceptibility is unclear, since *S*. *mansoni* does not directly involve the urogenital mucosa. This is in contrast to *S*. *haematobium* (uncommon in the Entebbe region [45]), which involves the urogenital mucosa and therefore alters mucosal HIV susceptibility directly at the site of sexual exposure [51–53]. However, *S*. *mansoni* infection does result in increased HIV target cells in the blood [54], and rhesus macaques infected with *S*. *mansoni* are 17-times more susceptible to SHIV [55].

Clinical trials of adult circumcision have demonstrated that the foreskin is a major site of HIV acquisition in uncircumcised heterosexual men [56–58]. We therefore sought to determine whether *S*. *mansoni* infection is associated with alterations in HIV target cells, both systemically, and at this relevant tissue site.

## Methods

### Study participants

Participants aged 18–49 years were recruited from the Uganda Virus Research Institute- International AIDS Vaccine Initiative (UVRI-IAVI) Voluntary HIV Counselling and Testing (VCT) Clinic in Kasenyi, Uganda, a fishing community on the shore of Lake Victoria. All men who expressed an interest in voluntary adult medical male circumcision were offered enrolment in the study. Exclusion criteria included current use of immunosuppressive therapy or infection with malaria. Men who had symptomatic genital infections were to be provided treatment and followed up weekly until resolution of symptoms, at which point they would be scheduled for circumcision. To avoid stigmatization, both HIV-infected and uninfected men were offered participation in this study, however, blood and foreskin samples were only analyzed for HIV-uninfected men. All participants were offered VCT, and HIV-infected men were referred to Wagagai Health Center (a private health facility at Kasenyi landing site) for treatment and care. Individuals who tested positive for schistosomiasis were treated at the clinic with single-dose praziquantel, 40 mg/kg body weight.

### Ethics statement

All participants provided written informed consent, and ethical approval was obtained through the Institutional Review Boards at the Uganda Virus Research Institute and at the University of Toronto.

### Sample collection and diagnostic testing

After VCT, consenting participants completed a behavioural questionnaire and were scheduled for circumcision. HIV testing was performed according to the Uganda National Algorithm, consisting of two rapid tests (Alere Determine HIV-1/2, Abbott Laboratories, Matsudo-shi, Japan; and HIV 1/2 STAT-PAK Dipstick Assay, Chembio Diagnostic Systems, Medford, USA), with a third rapid (Unigold HIV, Trinity Biotech, Wicklow, Ireland) used for discordant results. Participants returned to provide stool and terminal urine samples (collection vials from VWR, Mississauga, Canada) for three consecutive days surrounding surgery (i.e. the day prior to surgery, the day of surgery, and the day after surgery). Urine was immediately separated, with 1.5ml reserved for Circulating Cathodic Antigen (CCA) detection (stored at 4°C until analysis later the same day) and the remainder preserved with 0.2% bleach for *S*. *haematobium* egg detection by light microscopy [59]. Screening for *S*. *mansoni* was performed using the Kato-Katz method [59], with three slides screened per stool sample. Presence of urine-CCA, which does not distinguish between species of schistosomes and therefore cannot differentiate *S*. *mansoni* from *S*. *haematobium*, was detected using the Rapid Medical Diagnostics Schistosomiasis Test (Rapid Medical Diagnostics; Pretoria, RSA) according to the manufacturer’s directions. Previous studies in Rakai have found the Kato Katz method on two stool samples (sensitivity = 98.6%) or rapid CCA detection in one urine sample (sensitivity = 91.7%, specificity = 75.0%) accurately detect *S*. *mansoni* infection [60]. For the purposes of statistical comparisons in this study, men were classified as schistosomiasis-positive if they were positive by both Urine-CCA and had eggs in a stool sample (*S*. *mansoni*) on at least one study visit. Men were considered to be schistosomiasis-negative if negative for both Urine-CCA and eggs at all study visits.

Circumcisions were performed at the Wagagai Health Center at Kasenyi, Entebbe, Uganda. Prior to surgery, a clinical officer performed a physical examination and collected 16ml of whole blood. Surgery was deferred and participants were treated if urethral discharge or genital ulceration were present. Foreskins were collected into RPMI 1640 media supplemented with: 10% heat-inactivated FBS, 10U/ml penicillin, 10μg/ml streptomycin, 250ng/ml amphotericin B, and 2mM L-Glutamine (all from Gibco, Invitrogen; Carlsbad, CA, USA; henceforth referred to as R10 medium). Blood and foreskin samples were transported to UVRI-IAVI laboratories for immediate processing: four sections of foreskin were snap frozen into cryomolds in Optimal Cutting Temperature (OCT) compound (Fisher Scientific, Toronto, Canada) for immunohistochemistry (IHC); and one large section was reserved for T cell isolation. An aliquot of whole blood was removed for malaria diagnostics, and the remainder was separated into plasma and PBMC fractions by density gradient centrifugation (Ficoll-Paque Plus; Amersham Biosciences; Uppsala, Sweden). Participants were confirmed to be negative for malaria using both the Cypress Diagnostics Malaria falciparum Rapid Test (Langdorp, Belgium) and by microscopy (Giemsa stained thick smear). HSV-2 serology was performed by ELISA (Herpes Simplex Type 2 IgG ELISA, Kalon Biological Ltd., Guildford, UK), as previously validated in Uganda [61]. PBMCs were reserved for flow cytometry.

### T cell isolation from the foreskin

T cells were isolated from foreskin tissue as previously described [62]. Briefly, tissue was disrupted by a combination of mechanical and enzymatic digestion with 1.0ml of 500U/ml Collagenase Type I (Gibco, Invitrogen; Carlsbad, CA, USA) in the presence of 50U/ml of DNAse (Invitrogen). The resulting cell suspension was filtered through a 100μm cell strainer (BD Biosciences; Franklin Lakes, NJ USA) to remove any remaining undigested tissue. Filtered cells were allowed to rest under normal growth conditions (37°C, 5% CO_2_, humidified atmosphere) for 3–7 hours.

### Characterization of T cell subsets

PBMC and foreskin mononuclear cell counts were determined by trypan blue exclusion. 1x10^6^ PBMCs and 10-20x10^6^ foreskin mononuclear cells (depending on yield) were plated in 500μl culture medium with 5μg/ml Brefeldin A (GolgiPlug, BD Biosciences) and fluorochrome-labelled CCR6 antibody (11A9; BD Biosciences). Cells were stimulated for 9 hours at 37°C with either: 0.5ng/ml phorbol-12-myristate-13-acetate (PMA) and 0.5μg/ml ionomycin; 2μg/ml Staphylococcal enterotoxin B (SEB); or vehicle (0.1% Dimethyl sulfoxide, DMSO; all from Sigma; St. Louis, MO, USA). After stimulation, samples were washed with cold 2% Foetal Bovine Serum (FBS) in phosphate buffered saline (PBS) and stained with LIVE/DEAD Fixable Aqua Dead Cell Stain Kit (Life Technologies, Burlington, Canada), washed and then stained with fluorochrome-labeled monoclonal antibodies specific for the following surface antigens: CD3 Qdot655 (S4.1), CD4 PE-Cy7 (SK3), CCR5 PE-Cy5 (2D7/CCR5), CD69 APC-Cy7 (FN50), HLA-DR PE-CF594 (G46-6), and CD38 AF700 (HIT2; all BD Biosciences). In a second aliquot of cells, cells were stained for homing integrin α_4_ FITC (9F10), β_7_ APC (FIB504; both BD) and Cutaneous Lymphocyte Antigen (CLA) PE (HECA-452, Miltenyi Biotec; San Diego, CA, USA) in addition to CD3 and CD4. Samples were then washed and permeabilized using the eBioscience fixation/permeabilization solution (eBiosciences; San Diego, CA, USA) and stained with fluorochrome-labeled monoclonal antibodies specific for the following intracellular cytokines: IFNγ V450 (B27; BD Biosciences), IL17A AF488 (eBio64DEC17; eBioscience), and IL22 PE (22URTI; eBioscience). Data were acquired using an LSRII flow cytometer (BD Biosciences).

### CD3 density quantification by immunohistochemistry

All flow cytometry data were analyzed as relative proportions of CD3+ T cells. To determine if the overall density of CD3 T cells varied between men, as opposed to the relative abundance of specific subsets, the average number of T cells per mm^2^ of tissue was determined using IHC staining for CD3. OCT-cryopreserved tissues were sectioned to 5μm, fixed in acetone, and frozen for batch staining. At the time of staining, frozen sections were thawed and air-dried at room temperature. Sections were blocked with 10% normal goat serum and stained with rabbit anti-human CD3 antibody (Dako, Carpinteria, CA, USA) followed by goat anti-rabbit secondary antibody conjugated to AF647 (Life Technologies). Slides were then washed with PBS, counter stained with Hoechst DAPI, and air-dried. The number of CD3+ T cells per mm^2^ of tissue for each patient was derived from the average of four tissue sections: two from separate locations on the distal edge of the foreskin, and two from the proximal edge. A median of 6.10mm^2^ of foreskin tissue was analyzed by IHC per patient. Whole sections were scanned at 0.5μm/pixel using the TissueScope 4000 (Huron Technologies, Waterloo, Canada). Image analysis software (Definiens, München, Germany) was used to delineate the apical edge of the epidermis of the entire length of each section to a depth of 300μm (excluding artefacts or folds). CD3 cells were defined as nuclear DAPI fluorescence overlapping with, or directly adjacent to, AF647 fluorescence. The average density of CD3+ cells/mm^2^ of foreskin tissue was then multiplied by the percentage of CD3+ cells found to co-express CD4 by flow cytometry to calculate the average number of CD4 T cells per mm^2^ of foreskin tissue.

### Statistical analysis

Flow cytometry data was analyzed in FlowJo v.9.8.2 (Treestar; Ashland, OR, USA). Expression of surface markers was compared between Th subsets using the Friedman chi-square test; with post-hoc pairwise comparisons made using the Wilcoxon related samples rank test; Bonferroni adjusted p-values are reported. T cell populations were compared between *S*. *mansoni*-infected and uninfected men by Mann-Whitney U test. Analysis of Th17 cell polyfunctionality was performed using Boolean gating in FlowJo software, and polyfunctionality compared between *S*. *mansoni*-infected and uninfected men with a chi-squared distribution (1000 permutations of pies) using SPICE v5.35 software (Exon, National Institute of Allergy & Infectious Diseases)[63]. Post-hoc comparisons between functional Th17 subsets were made using related samples Wilcoxon rank test. Excel v.14.4.8 (Microsoft; Redmond, WA, USA) was used prior to statistical testing, and statistical tests were run using SPSS v.20.0 for Mac (IBM; New York, NY, USA). Based on previous foreskin T cell studies [26, 62, 64], a sample size of 34 men provided 80% power to detect a difference of one standard deviation in the proportion of either foreskin or blood CD4 T cells that were Th17 cells (α = 0.05).

## Results

### Study population

Participants consisted of 34 HIV-uninfected men living in fishing communities on the shores of Lake Victoria who were undergoing elective adult circumcision for HIV prevention. No participant tested positive for malaria, had a symptomatic genital infection, or was taking immunosuppressive therapy at the time of enrolment. As the immunopathology of schistosomiasis is the result of egg production, and not necessarily the presence of worms, we compared men with documented *S*. *mansoni* egg production on at least one of the three days surrounding surgery (n = 12; median 918 eggs per gram (epg); IQR 108–4644 epg) to control men who were free of both eggs and CCA in urine (an antigen originating from the gut of mature worms) for three consecutive days surrounding surgery (n = 12). No *S*. *haematobium* eggs were detected in any participant. Men who had detectable Urine-CCA but who were not shedding eggs (n = 10) were included graphically in the Th17 functional analysis, but were excluded from statistical analysis. *S*. *mansoni*-infected and control men were of similar age, HIV-risk taking behaviour (based on self-reported condom use, recent number of sexual partners, and transactional sex), and HSV-2 serostatus (Table 1). However, *S*. *mansoni* infection was associated with employment in the fishing industry (p = 0.04) and more frequent contact with lake water (p = 0.03).

**Table 1:** Participant demographics.

	*S*. *mansoni*		Uninfected		
	(n = 12)		(n = 12)		p-value
Age (yrs)	26.7	(18–37)	22.4	(18–31)	ns
HSV-2 Seropositive (%)	33.3		8.4		ns
Sex Partners Last 6 Months (#)	1.3	(0–3)	1.1	(0–4)	ns
Using Condoms					
Never	66.7		58.3		
Sometimes	8.4		16.7		ns
Always	25.0		25.0		
Works in the fishing industry (%)	58.3		0.0		0.037
Contact with lake > weekly (%)	58.3		8.4		0.027
Every treated for schistosomiasis (%)	16.7		8.4		ns

### Characterization of HIV target cell populations in the foreskin and blood

We first characterized Th1, Th17, and Th22 cells, independent of *S*. *mansoni* infection status, to test our assumption that these three Th subsets are preferential HIV targets. We examined expression levels of markers known to be associated with HIV susceptibility, namely the HIV co-receptor CCR5 [10, 65] and activation markers CD69 [65, 66] and HLA-DR [11, 66–68] on these Th subsets in the blood and foreskin of all 34 men.

**Fig 1:**
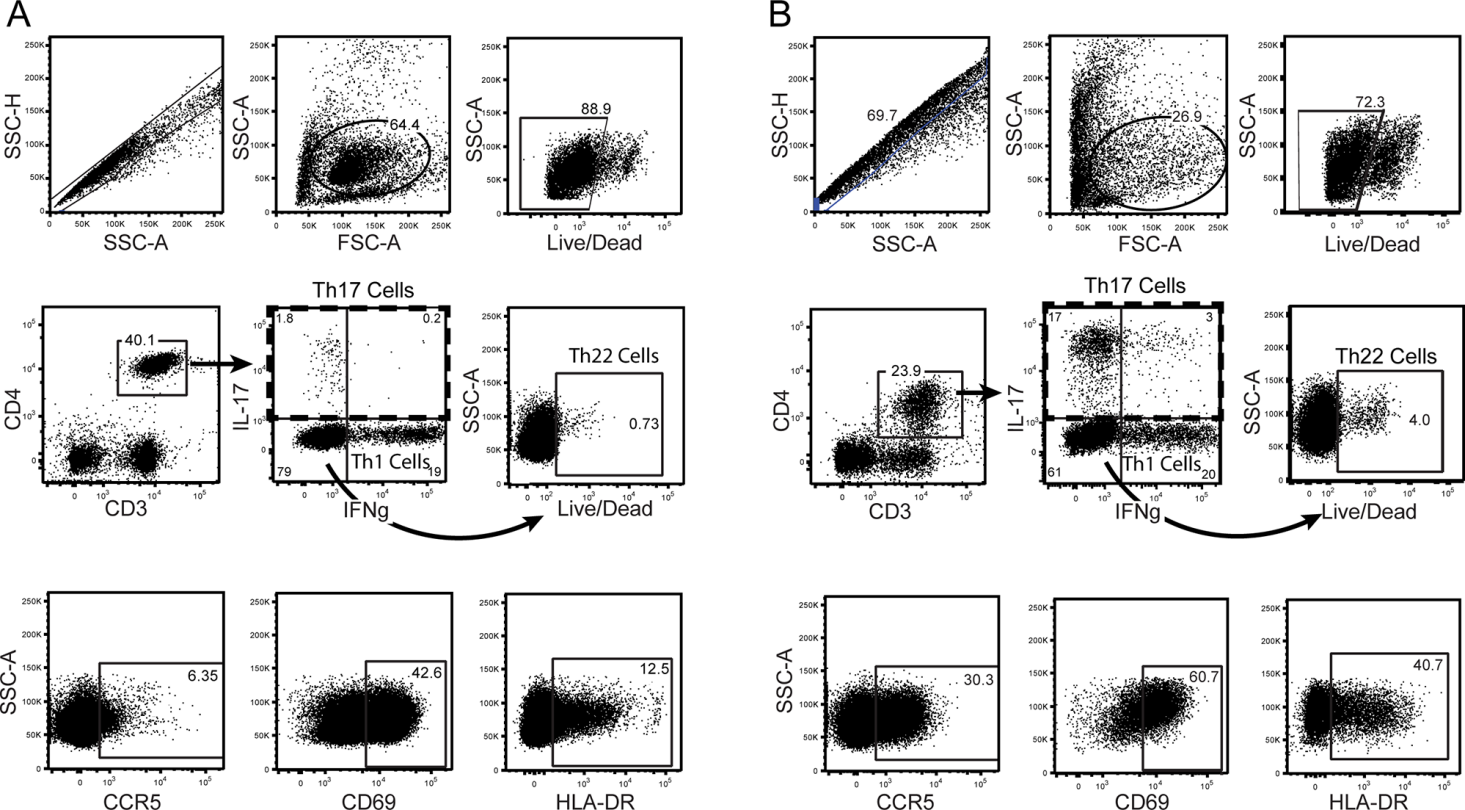
Flow cytometry gating strategy for Th subsets isolated from (A) the blood and (B) the foreskin. Blood cells were isolated by density centrifugation, and foreskin cells by mechanical and enzymatic digestion of tissue. Dead cells and doublets were excluded. Lymphocytes were identified based on characteristic size and granularity in blood samples and gates applied to foreskin samples. CD4 T cells were identified by expression of CD3 and CD4. Th subsets among CD4 T cells were identified by cytokine production in response to non-specific stimuli (SEB shown). Gates for cytokine production were based on unstimulated samples. Th17 cells were identified by production of IL-17A; Th1 cells by production of IFNγ and not IL-17A; Th22 cells by production of IL-22 in the absence of either IFNγ or IL-17A. Representative plots showing expression of CCR5, CD69 and HLA-DR on CD4 T cells are also shown.

CCR5 expression levels were decreased after PMA/iono stimulation (median 1.4% CCR5+ of CD4 T cells after PMA/iono vs. 3.2% on unstimulated), and so we report expression levels on functional subsets defined using SEB stimulation. Gating strategy and representative plots of stimulated cells are shown in Fig 1. Th subset definitions were mutually exclusive and exhaustive: Th17 cells produced IL-17A, and either IFNγ or IL-22; Th1 cells produced IFNγ, but not IL-17A; Th22 cells produced IL-22, but not IL-17A or IFNγ; and “other CD4 T cells” (the comparator group) were Th cells that did not produce any of these three cytokines upon stimulation (Fig 1).

**Fig 2:**
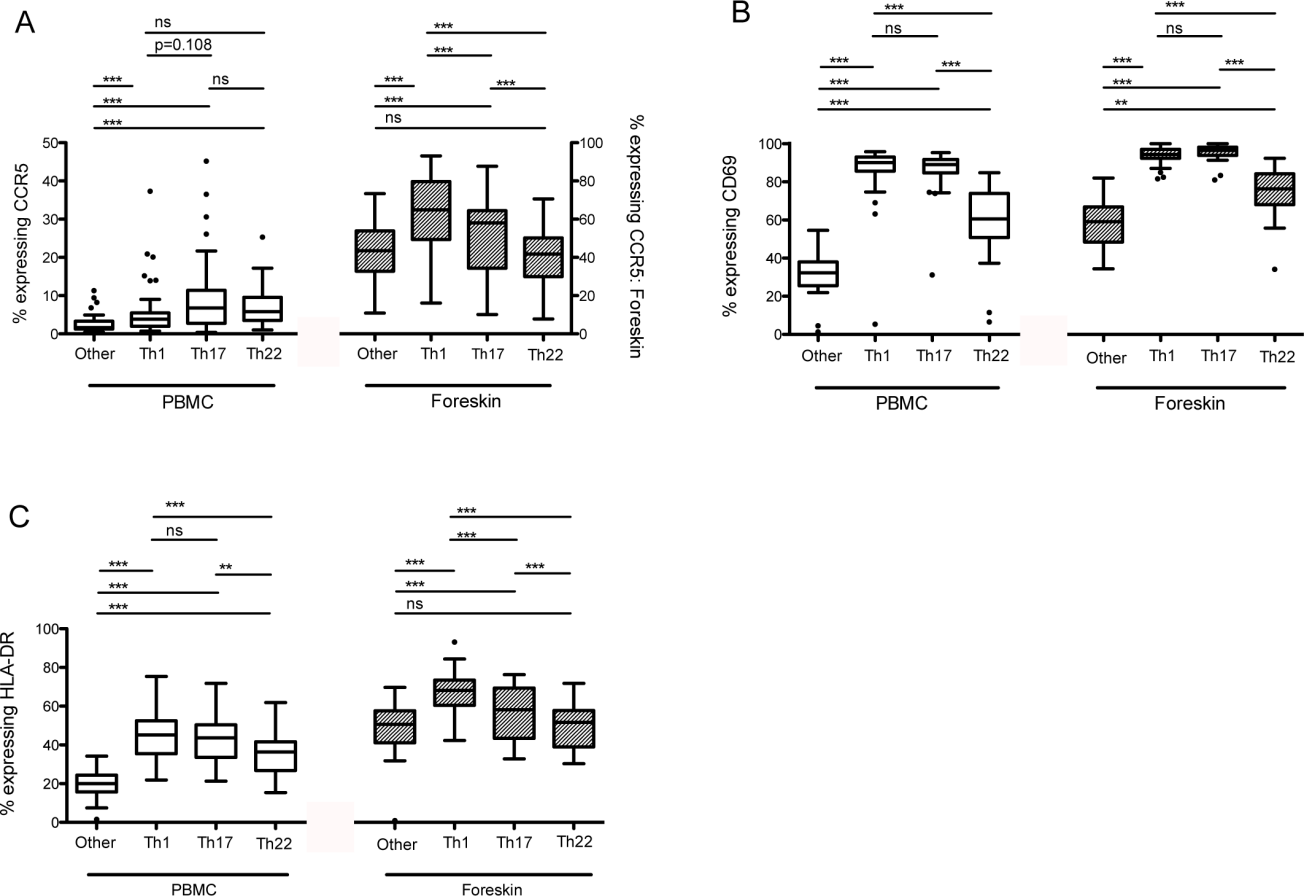
Differential expression of markers of HIV susceptibility on Th populations isolated from the blood and foreskin. Expression of (A) CCR5, (B) CD69, and (C) HLA-DR was measured on CD4 T cells isolated from the blood (PBMCs) and foreskin tissue using flow cytometry. “Other” refers to CD4+/CD3+ cells that do not produce IL-17A, IFNγ or IL-22. Expression of surface markers on Th subsets was compared using the Friedman chi-square test; post-hoc pairwise comparisons made using Wilcoxon related samples rank test and Bonferroni adjusted p-values are reported (*p<0.05; **p<0.01; ***p<0.001).

In the blood, all three Th subsets (Th1, Th17 and Th22) were more likely to express the HIV co-receptor CCR5 than other CD4 T cells (other CD4 T cells: 2.8%; all p<0.001; Fig 2A), although there were no significant differences between Th subsets (Th17: 10.8%; Th22: 7.1%; Th1: 6.3%). All three subsets also expressed higher levels of activation markers CD69 and HLA-DR compared to other CD4 T cells (other CD4 T cells: 31.1% CD69+, Fig 2B; and 20.0% HLA-DR+, Fig 2C; all p<0.001). Th17 and Th1 cells had substantially higher levels of both activation markers (87.9 and 86.0% CD69+, respectively; 43.4 and 45.9% HLA-DR+, respectively); Th22 cells were also more activated than other CD4 cells (60.5% CD69+ and 35.8% HLA-DR+, p<0.001), albeit less so that Th1 or Th17 cells (both p<0.001).

In the foreskin, Th1 and Th17 cells expressed significantly higher levels of CCR5 than other CD4 T cells (61.1% and 52.6% vs. 42.7%, both p<0.001; Fig 2A). In contrast to the blood, Th22 cells expressed no more CCR5 than other CD4 T cells (39.0 vs. 42.7%, ns). Also in contrast to the blood, foreskin Th1 cells expressed significantly more CCR5 than Th17 cells (p<0.001). Expression of HLA-DR on foreskin Th subsets followed a similar pattern, with Th1 cells expressing highest levels (69.4%; p<0.001 vs. other CD4 T cells and Th17 cells), followed by Th17 cells (59.1%, p<0.001 vs. other CD4 T cells), and Th22 cells having similar expression to other CD4 T cells (51.6% and 48.8%, respectively; Fig 2B). All three Th populations of interest had higher CD69 expression than other CD4 T cells (other CD4 T cells: 58.1%, all p<0.001; Fig 2B), with Th17 and Th1 cells having higher levels (both 94.0%) than Th22 cells (68.7%; p<0.001). In a separate flow cytometry panel, we assessed the expression of gut and skin homing markers on foreskin and blood CD4 T cells. We found the foreskin to be enriched for CD4 T cells expressing the skin homing marker CLA compared to the blood (59.8% of foreskin CD4 T cells vs. 18.2% of blood, p<0.001), but to have significantly reduced expression of the mucosal homing marker α_4_β_7_ (3.2% vs. 6.8%, p<0.001; S1 Fig).

### HIV target cell proportional frequency and absolute number in *S*. *mansoni*-infected and uninfected men

**Fig 3:**
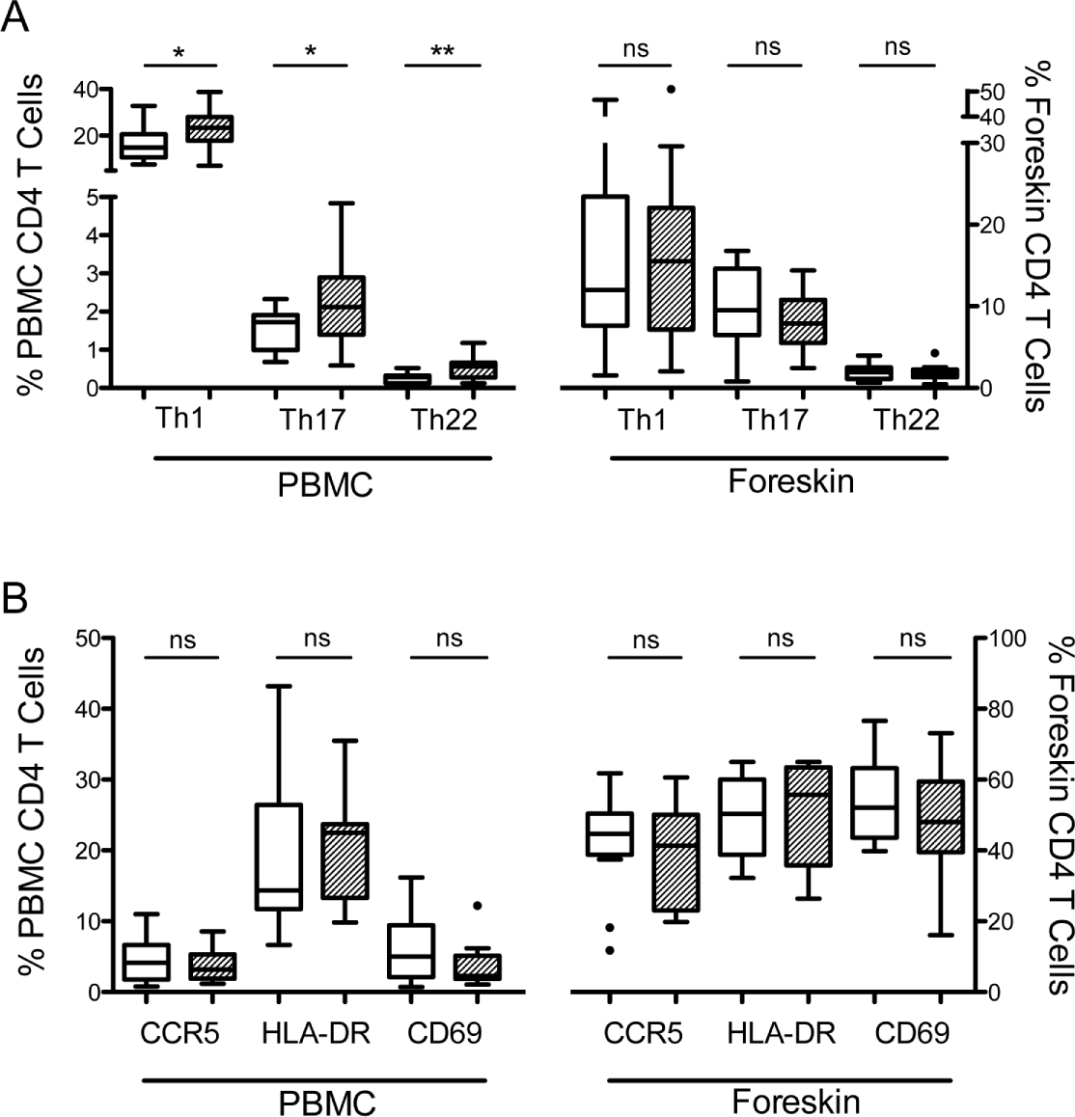
HIV target cells in the blood and foreskin of men infected with *S*. *mansoni*. HIV target cells were identified by flow cytometry from cells isolated from the blood (PBMC) or foreskin, and were compared between men shedding *S*. *mansoni* eggs (hatched bars) and men who were free of *Schistosoma* infection (clear bars). (A) Proportions of CD4 T cells that are Th1, Th17 or Th22 cells. (B) Expression of markers of HIV susceptibility on CD4 T cells. T cell populations were compared between *S*. *mansoni*-infected and uninfected men by Mann-Whitney U test (*p<0.05; **p<0.01).

*S*. *mansoni*-infected men had significantly higher frequencies of Th1 (22.9 vs. 16.5% of CD4 T cells, p<0.05; Fig 3A), Th17 (2.3 vs. 1.5%, p<0.05) and Th22 (0.5 vs. 0.3%, p<0.01) cells in their blood compared to uninfected men. However, these differences did not extend to the foreskin, since *S*. *mansoni*-infected and uninfected men had similar frequencies of susceptible Th populations in foreskin tissue. We also did not observe significant differences in overall CD4 T cell expression of CCR5, CD69 or HLA-DR between *S*. *mansoni-infected* and uninfected men, in either the blood or foreskin (Fig 3B). While flow cytometry provides the proportion (%) of CD3 T cells expressing markers of interest, it does not provide information on the total number of T cells present in the tissue. Using IHC to determine the number of CD3 T cells per mm^2^ of tissue, we found no statistically significant difference in the average density of foreskin CD3+ cells (262/mm^2^ (IQR 134-471/mm^2^) vs. 191/mm^2^ (IQR 125-533/mm^2^); p = 0.52) in *S*. *mansoni*-infected and uninfected men.

### Impact of *S*. *mansoni* infection on Th17 cell function

**Fig 4:**
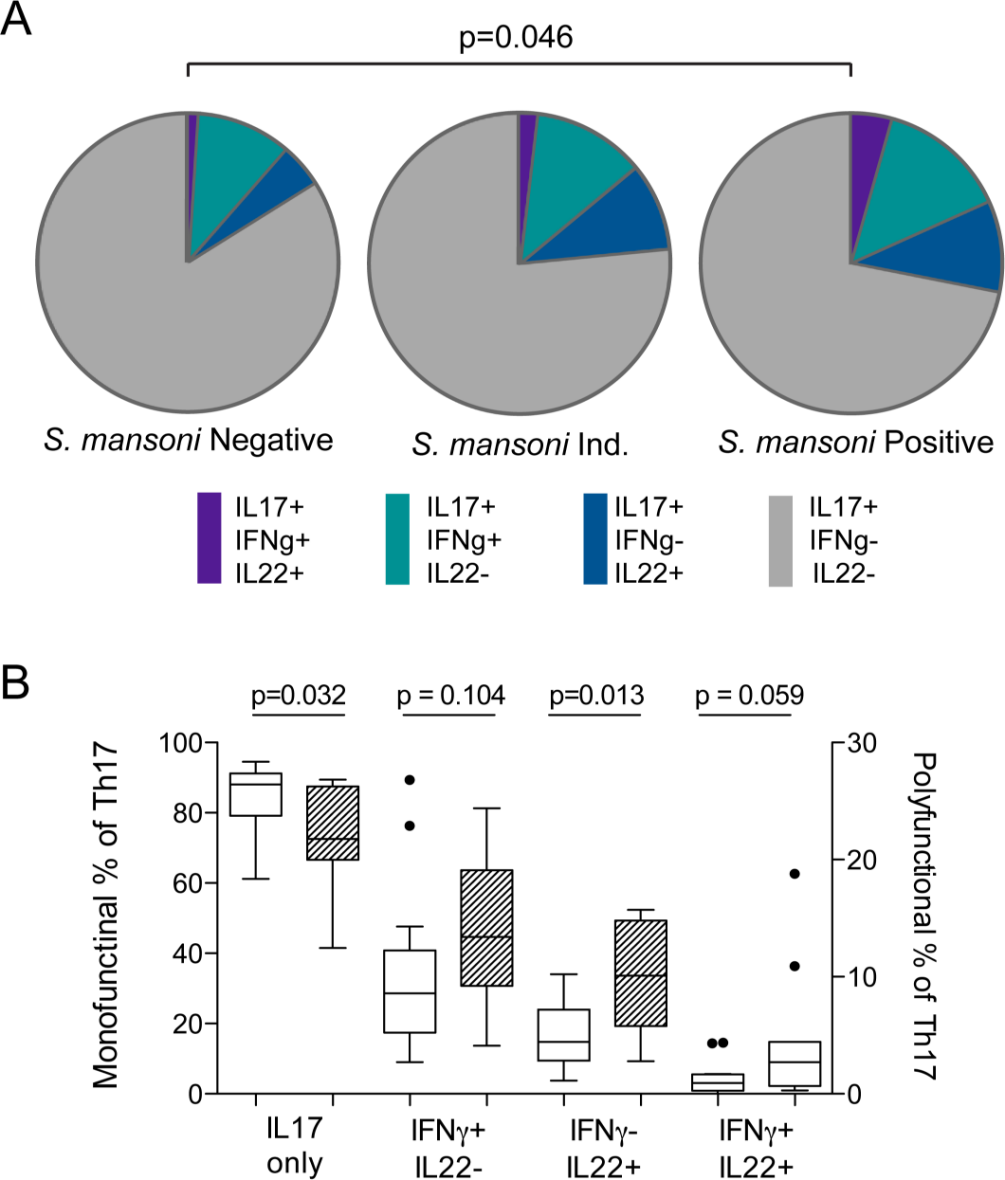
Functional capacity of Th17 cells isolated from the blood of men infected with *S*. *mansoni*. The ability of Th17 cells isolated from the blood to produce a second or third cytokine (IL-22 and/or IFNγ in addition to IL-17A) in response to non-specific stimulation (PMA/iono) was measured using flow cytometry. (A) Proportion of Th17 cells producing additional cytokines was compared between men infected with *S*. *mansoni* (shedding eggs), intermediate (presence of worms detected by Urine-CCA, but not shedding eggs), and uninfected men (no detection of worm antigen or eggs). Assessment of polyfunctionality was performed using SPICE v5.35 software with polyfunctionality compared between *S*. *mansoni*-infected and uninfected men using a chi-squared distribution (1000 permutations of pies)[63]. (B) Post-hoc comparison of individual functional subsets between *S*. *mansoni*-infected (hatched bars) and uninfected men (related samples Wilcoxon rank test).

We then went on to compare the functional capacity of Th17 cells, defined as their ability to co-produce IL-22 and/or IFNγ in addition to IL-17A, between *S*. *mansoni*-infected and uninfected men. Th17 cells from the blood of men infected with *S*. *mansoni* had a greater capacity to produce pro-inflammatory cytokines than those from uninfected men (Fig 4A, p = 0.046). Post-hoc analysis performing pairwise comparisons showed trends to increased numbers of triple cytokine producing cells (2.7 vs. 0.92%, p = 0.059) and IFNγ-producing Th17 cells (13.4 vs. 8.6%, p = 0.104), and significantly increased IL-22 producing Th17 cells (10.1 vs. 4.4%, p = 0.013; Fig 4B) in *S*. *mansoni*-infected men.

## Discussion

The likelihood of infection after sexual exposure to HIV is heterogeneous and likely determined by availability of susceptible CD4 T cells at the site of exposure [6]. Activated CD4 T cells [11–13] and Th1, Th17 [19–24], and Th22 [25] subsets have been implicated as preferential targets of HIV, and their frequency at mucosal sites may be altered by co-infections. In keeping with the hypothesis that these Th cell subsets represent preferential HIV targets, they demonstrated an elevated expression of the HIV co-receptor CCR5, and were more likely to express activation markers CD69 and HLA-DR, markers associated with increased HIV cellular entry [65]. Not only did we find that *S*. *mansoni* infection was associated with an increased systemic (blood) frequency of Th1, Th17 and Th22 cells, but functional analysis of blood Th17 cells demonstrated that *S*. *mansoni* infection was associated with increased production of pro-inflammatory cytokines IL-22 and IFNγ, a characteristic associated with selective depletion during HIV infection [25]. However, the systemic immune impact of *S*. *mansoni* infection did not extend to the foreskin. Specifically, we found no difference in the frequency of Th1, Th17 or Th22 cells, or in immune activation or CCR5 expression, between the foreskins of *S*. *mansoni-infected* and uninfected men. Of note, Th subsets in the foreskin had a different pattern of CCR5, HLA-DR and CD69 expression compared to blood, with Th22 cells at this site having lower CCR5 expression and activation status than Th1/Th17 subsets.

Our observation of increased systemic CD4 T cell activation is consistent with the known immunopathology of *S*. *mansoni*. A robust CD4 T cell response is essential for egg excretion during *S*. *mansoni* infection, demonstrated by the observations that egg excretion does not occur in immunocompromised mice [69, 70] or humans [50]. Eggs may also become trapped in the tissue on their way to being excreted [71], and the development of granulomatous lesions around them is dependent on CD4 T cells [72, 73]. While early studies in mice attributed the immunopathology of schistosomiasis infection to IFNγ production and Th1 activation [74, 75], the identification of Th17 cells as a distinct Th subset [76] led to the observation that Th17 cells were integral to granuloma formation, and high levels of IL-17A were produced by granuloma cells [77, 78]. Our findings of increased frequencies of Th1 and Th17 cells in the blood of *S*. *mansoni-infected* men is consistent with these previous observations. To our knowledge Th22 cells have not been studied in the setting of *S*. *mansoni* infection, but since they play a role in the maintenance of intestinal epithelial integrity [18, 25, 79] we hypothesize that the increase we observed during *S*. *mansoni* infection may be in response to the inflammatory foci that form in the intestinal epithelium during egg excretion.

Th17 and Th1 cells are highly susceptible to HIV *in vitro*, with IFNγ-producing Th17 cells (elevated in *S*. *mansoni-*infected men in the current work) being particularly susceptible [20–22], and Th22 cells may also be more susceptible [25]. However, the increased frequency of the highly susceptible Th subsets that we observed in the blood of *S*. *mansoni-infected* men was not observed in foreskin tissue. Since the foreskin is the primary site of HIV acquisition in heterosexual Ugandan men, the potential impact of these systemic *S*. *mansoni*-associated immune changes on HIV susceptibility in heterosexual men is not clear. This is in keeping with two recent publications that found no association between *S*. *mansoni* infection and either HIV incidence [80] or prevalence [81] in Entebbe, Uganda. However, our findings do emphasize the need for tissue specific studies when assessing the potential impact of co-infections on HIV susceptibility. This need was further demonstrated by the different patterns of CCR5 expression observed on Th subsets in the blood compared to foreskin: while blood CCR5 expression would suggest that Th22 cells were more HIV-susceptible than Th1 cells, foreskin Th22 cells had relatively low CCR5 expression.

We had hypothesized that *S*. *mansoni* infection would alter HIV target cells in the foreskin through activation of the common mucosal immune system. Local gut epithelial damage occurs during *S*. *mansoni* egg excretion and would be expected to directly increase the frequency of HIV target cells at this site, likely explaining the increased susceptibility to SHIV seen in *S*. *mansoni*-infected primates after rectal but not systemic virus challenge [55]. However, we had speculated that the upregulation of common mucosal T cell homing molecules, such as the integrin α_4_β_7_ [82, 83], might also increase T cell activation and HIV target cell numbers at genital mucosal sites. One reason that we did not observe this may be that T cells in the foreskin expressed high levels of the skin homing marker CLA, but not α_4_β_7_.

This study had two limitations that should be considered. The first is the relatively small sample size. This study was designed to determine if a larger, prospective study of the effect of *S*. *mansoni* infection on foreskin immunology was warranted. It was powered to detect a difference of one standard deviation in Th populations between groups, and so more subtle chances in foreskin T cell immunology would be missed. However, the robust immune differences seen in the blood without consistent trends in the foreskin suggest that a larger sample size would not change our conclusions. A second limitation of this study was the lack of diagnostic testing for STIs other than HSV-2. Genital co-infections are associated with local mucosal immune activation [33], and therefore if they were overrepresented among control men (*S*. *mansoni*-uninfected), this would have contributed to the absence of *S*. *mansoni*-associated foreskin immune changes in this study. However, since there was a non-significant trend to increased HSV-2 prevalence in *S*. *mansoni*-infected men, it seems unlikely that these same men had significantly lower rates of other STIs. Furthermore, since HSV-2 infection is associated with T cell activation in the foreskin but not blood [35], it is not likely that inter-group differences in STI prevalence explain the presence of T cell immune alterations in the blood.

In conclusion, we found that *S*. *mansoni* infection in HIV-uninfected Ugandan men was associated with an increase in highly HIV-susceptible T cell subsets in the blood, but not the foreskin. Whether these immune changes would alter HIV susceptibility, given that they are limited to the blood compartment, is not clear. Future studies are warranted to determine if the systemic immune changes observed in this study extend to other mucosal sites of HIV acquisition, such as the cervicovaginal or gut mucosae.

## Supporting Information

S1 Checklist STROBE checklist. (DOC)

S1 Fig Expression of homing markers on CD4 T cells isolated from the blood and foreskin. (A) Representative staining and (B) expression of Cutaneous Lymphocyte Antigen (CLA) or integrin α_4_β_7_ on CD4 T cells isolated from either the blood (clear bars) or foreskin tissue (hatched bars). Homing marker expression was compared between foreskin and blood cells by Wilcoxon related samples rank test (**p<0.01).
(TIF)
