# Supplementary figures and images for: Schistosoma mansoni Infection in Ugandan Men Is Associated with Increased Abundance and Function of HIV Target Cells in Blood, but Not the Foreskin: A Cross-sectional Study
Source: PLoS Negl Trop Dis. 2015 Sep 3;9(9):e0004067. doi: 10.1371/journal.pntd.0004067 (PMC4559468; doi:10.1371/journal.pntd.0004067)

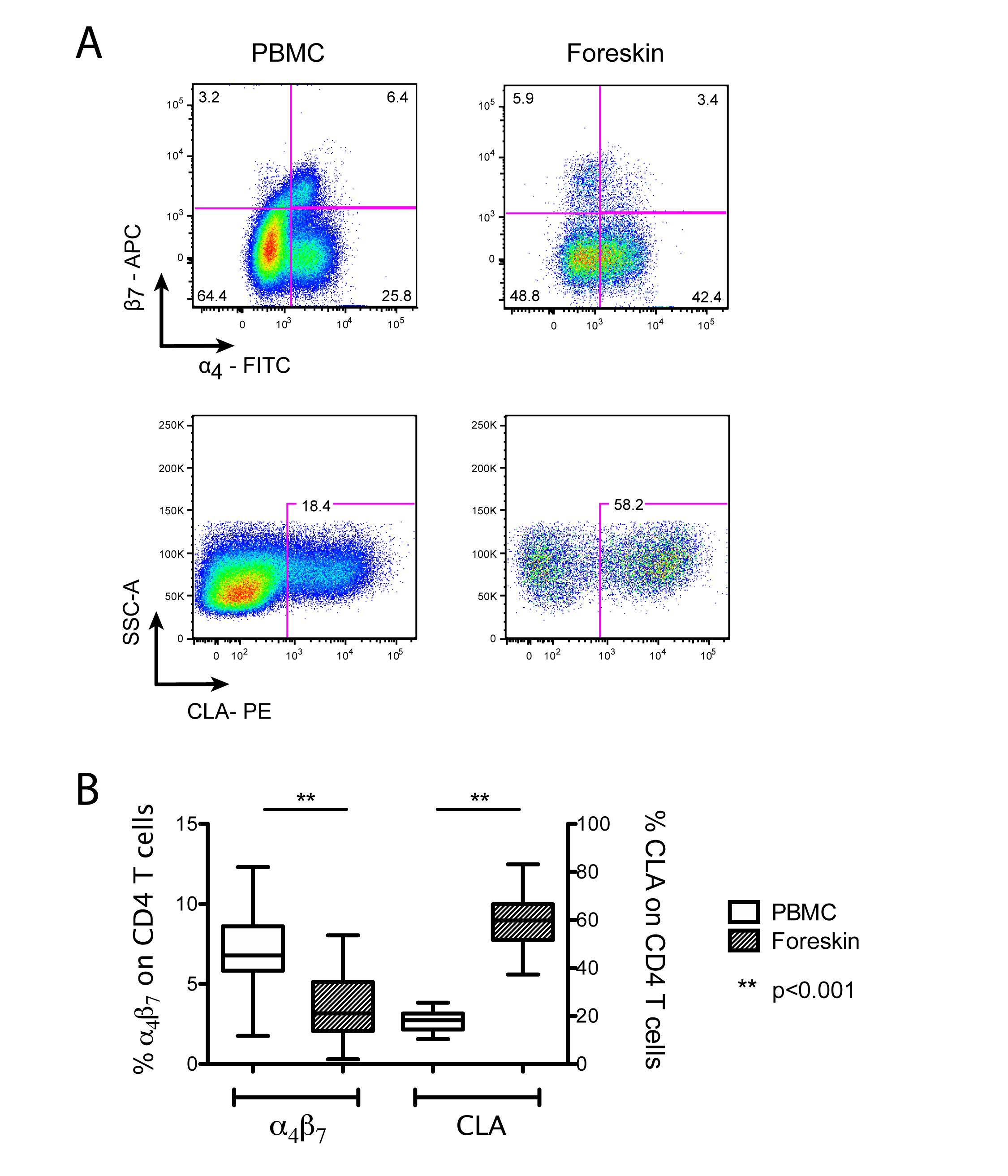

Supplement: S1 Fig — (A) Representative staining and (B) expression of Cutaneous Lymphocyte Antigen (CLA) or integrin α4β7 on CD4 T cells isolated from either the blood (clear bars) or foreskin tissue (hatched bars). Homing marker expression was compared between foreskin and blood cells by Wilcoxon related samples rank test (**p<0.01). (TIF) [file pntd.0004067.s002.tif]
